# Inherent and Stress-Induced Responses of Fine Root Morphology and Anatomy in Commercial Grapevine Rootstocks with Contrasting Drought Resistance

**DOI:** 10.3390/plants10061121

**Published:** 2021-06-01

**Authors:** Idan Reingwirtz, Jake Uretsky, Italo F. Cuneo, Thorsten Knipfer, Clarissa Reyes, M. Andrew Walker, Andrew J. McElrone

**Affiliations:** 1 Department of Viticulture and Enology, University of California, Davis, CA 95616, USA; ireingwirtz@ucdavis.edu (I.R.); juretsky@ucdavis.edu (J.U.); clareyes@ucdavis.edu (C.R.); awalker@ucdavis.edu (M.A.W.); 2 Faculty of Agriculture and Food Sciences, Pontificia Universidad Católica de Valparaíso, Valparaíso 2340025, Chile; italo.cuneo@pucv.cl; 3 Faculty of Land and Food Systems, University of British Columbia, Vancouver, BC V6T 1Z4, Canada; thorsten.knipfer@ubc.ca; 4 Crops Pathology and Genetics Research Unit, United States Department of Agriculture, Agricultural Research Service, Davis, CA 95616, USA

**Keywords:** fine root diameter, root populations cortical lacunae, fine roots, specific root length

## Abstract

Some grapevine rootstocks perform better than others during and after drought events, yet it is not clear how inherent and stress-induced differences in root morphology and anatomy along the length of fine roots are involved in these responses. Using a variety of growing conditions and plant materials, we observed significant differences in root diameter, specific root length (SRL) and root diameter distribution between two commonly used commercial grapevine rootstocks: Richter 110 (110R; drought resistant) and Millardet et de Grasset 101-14 (101-14Mgt; drought sensitive). The 110R consistently showed greater root diameters with smaller SRL and proportion of root length comprised of fine lateral roots. The 110R also exhibited significantly greater distance from tip to nearest lateral, longer white root length, and larger proportion of root length that is white under drought stress. Mapping of fine root cortical lacunae showed similar patterns between the rootstocks; mechanical failure of cortical cells was common in the maturation zone, limited near the root tip, and increased with drought stress for both genotypes; however, lacuna formed under wetter soil conditions in 110R. Results suggest that drought resistance in grapevine rootstocks is associated with thick, limitedly branched roots with a larger proportion of white-functional roots that tend to form lacuna under more mild water deficit, all of which likely favor continued resource acquisition at depth.

## 1. Introduction

Rootstocks are utilized in the production of a wide range of perennial and annual crop species to mitigate against deleterious biotic and abiotic factors and promote beneficial horticultural traits [1,2]. Commercially available grapevine rootstocks confer differential resistance to stresses that include drought, salinity, phylloxera, nematodes, and viruses. Pairing the proper rootstock with vineyard location is crucial for optimal grafted plant performance and, ultimately, fruit and wine quality [3,4]. Root traits are a critical component of rootstock effects on scions, since rootstocks only contribute roots and a small portion of the trunk to grafted vines. Characterizing morphological and anatomical traits related to drought resistance in grapevine roots could facilitate more rapid germplasm screening for rootstock breeding.

Water acquisition and response to drought involve complex relationships among root ontogeny, physiology, anatomy, and morphology. In woody perennials like grapevine, root genesis, maintenance, and turnover also affect plant-environment interactions [5]. Woody root systems are generally described as populations of suberized coarse roots (i.e., >2 mm) and fine roots (i.e., <2 mm) that are either suberized or unsuberized [6,7]. Water flows preferentially through unsuberized fine roots [6,8,9] compared to suberized coarse roots with low radial hydraulic conductivity [6,10,11]. In grapes, these two broad classes correspond with young lateral roots and the perennial roots from which they emerge [12]. Clonally propagated grapevines do not possess primary taproots, but instead produce several adventitious roots that eventually form the structural foundation of the perennial root system [13,14] and are analogous to post-embryonic axial roots of crops grown from seed. Several orders of perennial lateral roots can develop from these primary roots, acting as scaffolding for the short-lived fine roots that are so critical in resource acquisition [13].

Although generalizations of root function are made based on diameter, there is high variance in absolute diameter among taxa, and functionality and lifespan of individual roots are often related to their developmental stage and relative position within the root system (i.e., root order) [15,16]. For example, a previous study reported that root order corresponded with differences in root diameter, tissue density, specific root area, and water flux density and efficiency in Volkamer lemon citrus rootstock (*Citrus volkameriana* Ten. and Pasq.) [17], with first order roots measuring approximately 0.5 mm diameter and the highest order roots exceeding 4.5 mm diameter. In extremely fine-rooted plants like highbush blueberry (*Vaccinium corymbosum* L.) that possess higher-order roots less than 1 mm diameter, root order is also closely associated with diameter, lifespan and traits affecting functionality [18]. Specific root length (SRL; root length ⋅ dry biomass^−1^) is the ratio of root length to biomass and is used to index efficiency of nutrient acquisition versus utilization in roots [19,20]. However, comparisons of SRL among taxa are difficult due to differences in absolute root diameter among taxa like those described previously [5]. Nonetheless, the relationships among root order, root diameter and SRL reflect the general distribution of roots of different functional classes within root systems [16], especially the relative proportion of absorptive fine roots, and might indicate root architectural traits that impact resource acquisition [21,22].

Functionally, unsuberized fine roots are critically important for plant function as they are responsible for the vast majority of water absorption [6,8,9]. Even though there have been recent efforts to improve fine root classification based on form and function [15], the current understanding of stress-induced shifts in root function remains vague. For example, a previous study showed that first-order roots of *Citrus* had significantly higher water uptake rates than second and third-order roots under optimal conditions, but this relationship flipped when citrus root systems were subjected to salinity stress [17]. During drought stress, root systems may respond differently by species or genotype. Root growth increases in some species and genotypes during periods of decreased water availability [23,24], while others reduce total root growth [25]. Alsina et al. (2011) found that high hydraulic conductance in a drought-resistant grapevine rootstock was linked to maintenance of fine root proliferation late in the growing season that was not observed in a drought-sensitive rootstock [26]. From an anatomical perspective, Zhu et al. (2010) found that lacunae formation in fine root cortical cells increased the drought tolerance in corn plants, presumably by favoring the root tip over other parts of the root system [27]. Recently, similar results were reported for grapevine rootstocks where root tip function during recovery (i.e., for re-watered plants after drought) increased in roots that showed more lacunae formation [28,29]. However, the exact position of lacunae formation along the length of grapevine fine roots and whether this phenomenon is associated with root development or drought remain unknown.

The commonly used grapevine rootstocks 110R (*Vitis berlandieri* × *V. rupestris*) and 101-14Mgt (*V. rupestris* × *V. riparia*) have been described as drought resistant and drought sensitive, respectively [3,4,30]. In the present study, we examined root morphology of the two rootstocks in plants grown from herbaceous cuttings in the greenhouse and in field plots. We then performed a rhizotron experiment to explore morphological and anatomical traits by mapping lacunae presence along the length of fine roots of the two rootstocks when subjected to drought. Our primary objective was to study differences in fine root production between the cultivars at different growth stages, and in mapping lacunae formation in different positions associated with different soil water content.

## 2. Results

### 2.1. Root System Morphology

**Figure 1:**
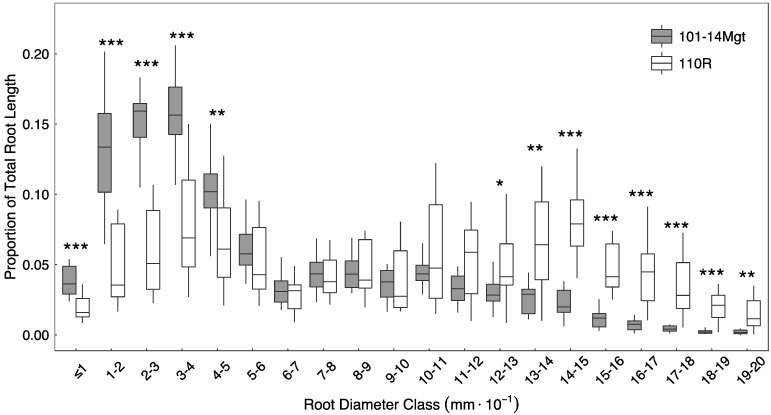
Root distributions by diameter class in 101-14Mgt and 110R root systems grown from herbaceous cuttings in the greenhouse (*n* = 15). Boxes represent first and third quartiles; whiskers represent samples within 1.5 times the interquartile range. Although included in statistical analyses, outliers are not displayed. Asterisks indicate significant differences between rootstocks at each diameter class as determined by Welch’s *t*-test. *p* < 0.05 = *; *p* < 0.01 = **; *p* < 0.001 = ***.

We assessed root system morphology in 110R and 101-14Mt rootstocks from varied plant materials and under a variety of growing conditions. Regardless of the conditions, root system morphology was consistently different between the rootstocks (Table 1 and Table 2, Figure 1 and Figure S1). For both herbaceous cuttings grown in the greenhouse and dormant woody cuttings grown in the field, mean root diameters were consistently larger for 110R compared to those of 101-14Mgt (*p* < 0.001 for both greenhouse and field grown vines (Table 1)). These differences in root diameter between rootstocks also corresponded with consistently lower SRL in 110R roots compared with 101-14Mgt roots (Table 1). Closer inspection of the root diameter distributions (Figure 1 and Figure S1) shows that 101-14Mgt has a larger proportion of total root length represented by the smallest diameter classes. Roots of 101-14Mgt between 0–0.5 mm diameter accounted for 58.8% of total root length, while only 2.9% of the total root length was made up by roots in the 1.5–2.0 mm diameter class (Figure 1). In 110R, 0–0.5 mm and 1.5–2.0 mm diameter roots accounted for 27.3% and 16.6% total root length, respectively. In fact, 48.2% of total root length comprised roots 1.0–2.0 mm diameter in 110R, compared to only 18.6% in 101-14Mgt (Figure 1). These morphological patterns were consistent for field grown woody cuttings harvested after 1 and 2 years of growth (Figure S1).

In order to better understand the root morphological and anatomical responses of the rootstocks to drought stress, we constructed custom-made rhizotrons to track morphological and anatomical changes. Consistent with morphological assessments described above, mean root diameter was significantly greater in 110R than 101-14Mgt under control conditions (1.91 and 1.21 mm, respectively), and maintained this trend under drought (1.55 and 1.22 mm, respectively; Table 2). The 101-14Mgt had significantly greater total root length (TRL) and number of laterals under drought, while 110R showed significantly greater distance from tip to nearest lateral, higher white root length, and higher proportion of root length that is white under drought conditions.

### 2.2. Cortical Lacunae Mapping

**Figure 2:**
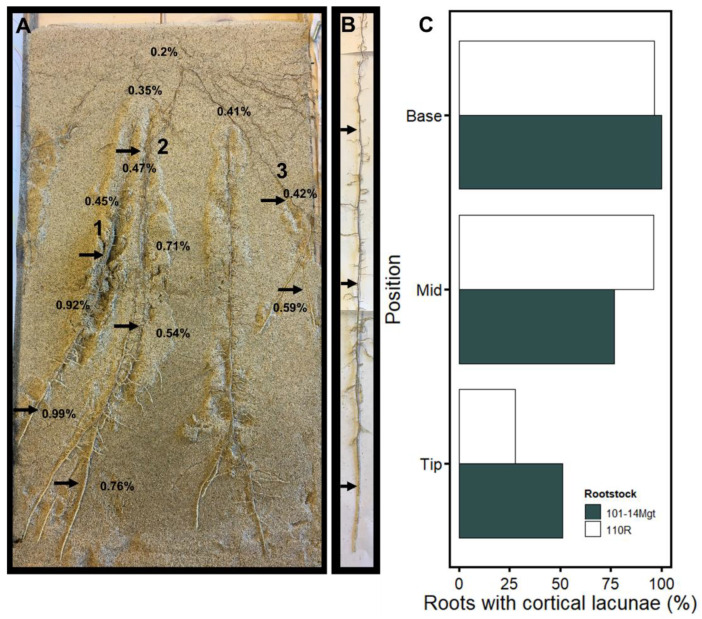
Lacunae mapping along the length of 101-14Mgt and 110R fine roots. Black arrows in panel (**A**) indicate the location of the scanning in three roots associated with a specific soil water content (%) collected adjacent to the root. The extracted root in (**B**) correspond to root 1 in in (**A**). In (**C**), percentage of root showing cortical lacunae in the root base, mid-section, and tip corresponding to the position showed by arrows in (**B**).

We mapped cortical lacunae along the length of grapevine fine roots of 101-14Mgt and 110R in plants in the custom rhizotrons (see example root systems in Figure 2). Root growth rate (mm/day) was significantly higher under well-watered conditions (control; *p* = 0.0001; 21.2 mm day^−1^ ± 0.4 SE for well-watered and 15.7 mm day^−1^ ± 1.7 SE for drought), yet no differences were found between cultivars. Percentage of fine roots exhibiting cortical lacunae was higher in the base (96.6% in 110R and 100% in 101-14Mgt), followed by the mid-section (96.2% in 110R and 76.9% in 101-14Mgt), and finally, the root tip (27.9% in 110R and 51.2% in 101-14Mgt) (Figure 2C).

**Figure 3:**
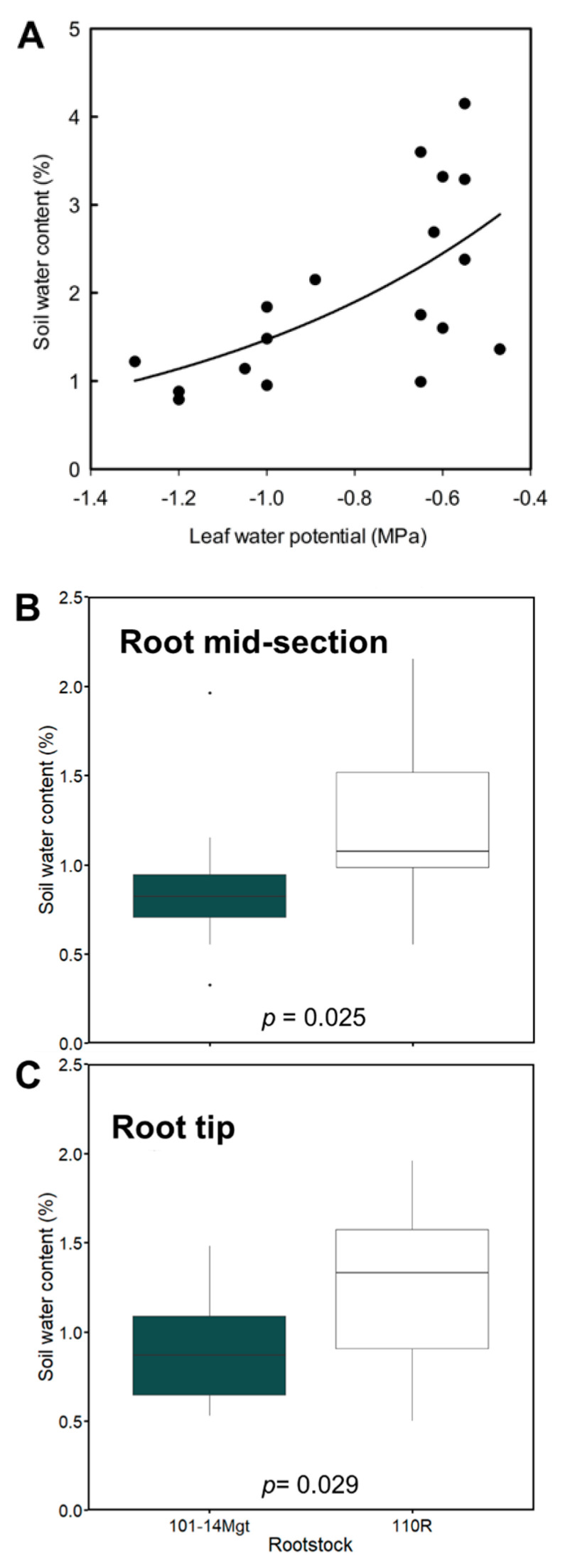
(**A**) Relation between soil water content (%) and stem water potential (MPa) (solid line is best fit, *y* = 5.27 ∗ exp^(1.27x)^, R^2^ = 0.37, *p* = 0.006). In (**B**,**C**), soil water content (%) associated with the presence of cortical lacunae in the root mid-section (**B**) and root tip (**C**). *p*-values in (**B**,**C**) were determined using *t*-test.

**Figure 4:**
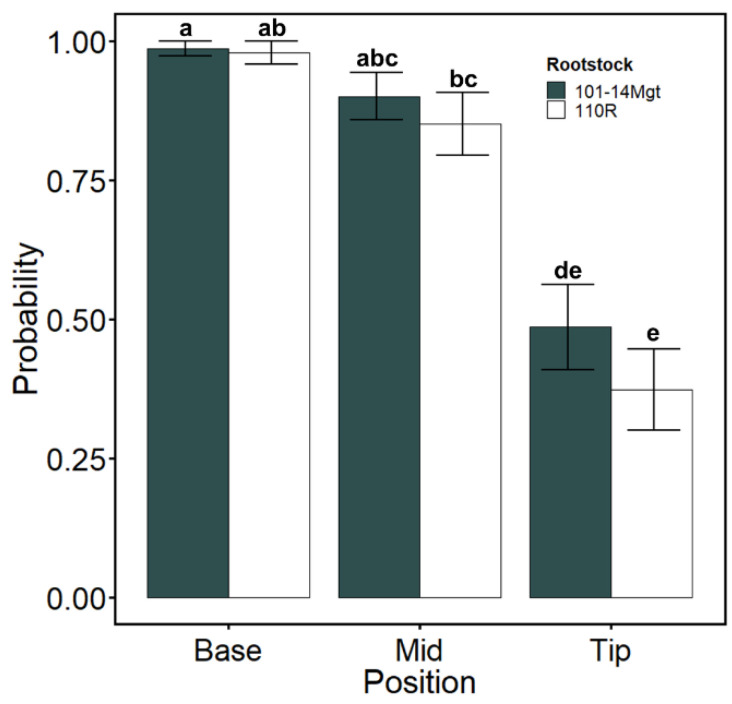
Estimated means probabilities of lacunae formation. Data are means ± SE. Different letters indicate significant differences with 95% confidence level.

To approximate water stress accurately, we measured soil water content (%) in the soil environment surrounding the position where lacunae were mapped (Figure 2A), and we found an exponential relation between soil water content (%) and stem water potential (MPa; Figure 3A). Soil water content (%) when cortical lacunae were present was significantly lower in 101-14Mgt in the mid-section (*p* = 0.025; Figure 3B) and in the root tip (*p* = 0.029; Figure 3C). Based on a logistic model, we found that the estimated means probabilities of lacunae presence were larger in the base of the root for both rootstocks (mean of 0.98 ± 0.017 SE; Figure 4). In contrast, the probability of lacunae presence was lower in the root tip for both rootstocks (mean of 0.43 ± 0.08 SE; Figure 4). In the mid-section of the root, the probability of lacunae presence was larger than in the tip averaging 0.87 ± 0.05 SE across rootstock and irrigation conditions (Figure 4). According to the classification table, the model was accurate in 78.3% of the cases (Table 3). In 15.3% of the cases, the model predicted lacunae presence when the observation was negative (false positive), while in 6.3% of the cases, the model predicted no lacunae presence when the observation was positive (false negative, Table 3).

## 3. Discussion

In this study, we characterized fine root morphology and study the presence of cortical lacunae along the length of grapevine fine roots to better understand how 110R and 101-14 Mgt, commercial rootstocks with contrasting drought resistance, are inherently different and respond to water stress. Our analysis of root system morphology revealed that 110R root systems have larger mean root diameter and lower SRL than 101-14Mgt roots across growth conditions and developmental stages observed in the study. The data presented here strongly suggest a trend toward maintaining existing roots in 110R but continued initiation of new roots in 101-14Mgt.

Grapevine roots show plasticity for growth and development depending on environmental conditions [31,32], and evidence exists for significant rootstock–scion interaction effects on root growth [33]. In our study, however, 110R root systems consistently possessed thicker roots with proportionally fewer fine roots than 101-14Mgt. Although generalizations of root function are made based on diameter, there is high variance in absolute diameter among taxa, and functionality and lifespan of individual roots are often related to their developmental stage and relative position within the root system [15,16]. While we did not distinguish among root orders directly, these data strongly suggest reduced branching and/or branch length in 110R than in 101-14Mgt. Such differences in root morphology might significantly affect root lifespan, function and response to environmental conditions. Several studies have shown strong relationships between root diameter and lifespan within [18,34,35] and across [36] woody species. Root diameter has also been positively correlated with elongation rate [37] and soil penetration [38] in monocotyledonous species. Moreover, root diameter has been positively correlated with cortex thickness, stele diameter, root length and vessel diameter, but negatively correlated with root branching in a collection of tropical and subtropical angiosperm species [39]. Root systems consisting of a limited number of thick, deeply penetrating axial roots with reduced lateral root density (i.e., low SRL) have been proposed as advantageous in acquiring mobile resources like water and nitrate. This has been attributed to reduced competition among roots, lower metabolic and respiratory costs, and concentration of root growth in deeper soil regions that maintain more stable moisture levels [40]. This ideotype has been validated with observations of recombinant inbred maize genotypes in which drought performance was negatively correlated with axial root number [41] and lateral branching density [42]. Similarly, the seedling roots of woody species indigenous to low rainfall environments display less branching and lower SRL than those of species found in wetter environments [43,44]. In the present study, the trend toward greater resource investment in existing roots in 110R than in 101-14Mgt could relate to robustness of the respective root systems over time, as well as the likelihood of retaining an overall root structure poised to resume growth and resource acquisition after periods of scarcity [29].

The two rootstocks observed in the present study were obtained from crosses between *V. riparia* × *V. rupestris* (101-14Mgt) and *V. berlandieri* × *V. rupestris* (110R), and ecological differences among the parental species offer insight into differences in root morphology and drought response observed between the cultivars. *Vitis riparia* is widely dispersed, growing in moist sandy soils across most of the eastern United States and southeastern Canada [45] and in close association with permanent sources of water [3,46]. *Vitis rupestris* is mostly restricted to the Ozark plateau, spanning between Missouri and Arkansas [47], in very gravelly streambeds proximal to visible water [46]. *Vitis berlandieri* is endemic to the Edwards Plateau in south-central Texas, which is characterized by shallow, limestone-derived soils and inconsistent precipitation [46,48]. We suspect that differences in root traits between 101-14Mgt and 110R result from differences in environmental adaptation of the respective parental species, particularly *V. riparia* and *V. berlandieri*. The fibrous root system and limited response to mild drought stress in 101-14Mgt reflect adaptation to consistently available moisture in the natural habitat of *V. riparia.* Conversely, the coarse root system and heightened initial drought response in 110R suggest adaptation to an environment in which water is only intermittently available, such as the Edwards Plateau in the case of *V. berlandieri*, where water acquisition from deeper sources would likely be very important [49,50].

Fine root cortical lacunae have been reported to modify the internal structure and hydraulic properties of unsuberized fine roots of monocots [51,52], desert succulents [53,54,55], and recently in grapevine [28,29]. However, most of these studies explored lacunae formation in specific locations along the length of fine roots, usually the maturation developmental region [28,29]. In a recent study, questions arose related to possible differential formation of cortical lacunae along the length of fine roots and the need to differentiate stress-driven formation of lacunae with developmental-driven collapse of the cortex. In here, our results highlight that lacunae are almost always present in the base for both rootstocks, which is interpreted here as a developmentally driven collapse of the cortex. Also, the mid-section position showed lacunae presence in a high proportion of roots. We observed lacunae in fine roots of 110R in the tip and mid-sections under wetter soil conditions. This suggests that 110R forms these under relatively mild water stress conditions, similar to findings in Cuneo et al. 2021. Overall, these results highlight that stress-driven lacunae formation tend to occur more in the transition between the meristematic/elongation and maturation zones, as previously reported [28,29], and provide further evidence that lacunae formation might be a physiological mechanism present in plants to maintain hydraulic connectivity and functionality of the root tip for resource acquisition [29]. The fact that rootstock was a weak predictor in the logistic model for lacunae presence suggests that cortical lacunae might be a trait related to shared alleles inherited from common ancestor *Vitis rupestris*. Future studies should include pure *Vitis rupestris* rootstocks to better understand the eco-physiological nature of cortical lacunae and, in this way, better understand the role of lacunae in native habitats.

## 4. Materials and Methods

### 4.1. Plant Material, Greenhouse Experiments

Plants of Richter 110 (110R; *Vitis berlandieri* × *V. rupestris*) and Millardet et de Grasset 101-14 (101-14Mgt; *V. riparia* × *V. rupestris*) were propagated from either herbaceous cuttings collected from mother plants in the University of California, Davis vineyards or dormant woody cuttings. The basal node of each cutting was soaked in 2.5% rooting solution (Earth Science Products, Wilsonville, OR, USA), placed in a plastic tray filled with perlite, and maintained in a fog room for ~15 days until root initiation and growth [56].

For root morphology observations, herbaceous cuttings were transplanted to square pots (7 cm × 7 cm × 23 cm) and custom-made chambers (rhizotrons of 71 cm × 3.75 cm × 2 cm) filled with sand and set in a greenhouse with natural lighting (~14/10 light/dark) and temperatures ranging between 25–30 °C. Plants were watered with modified Hoagland’s solution [8]. When roots were first observed emerging from the bottom of pots (~4 weeks), the root systems of all plants were removed from pots, washed, and placed in plastic bags for analysis. Roots that were not immediately analyzed were stored at ~2 °C with adequate moisture to prevent desiccation. In the case of the rhizotron experiment, we analyzed root morphology over time during a dry-down imposed by restricting irrigation and compared this to well-watered control plants [28,29].

### 4.2. Plant Material, Field Experiment

For the field experiment, dormant woody cuttings of 110R and 101-14Mgt were collected from plants in the University of California, Davis vineyards, soaked in water, and placed in a cold room at ~2 °C for nine days. *V. vinifera* winegrape cultivar Cabernet Sauvignon (CS) scion were grafted to 110R and 101-14Mgt rootstocks using an omega-punch grafting machine (Fornasier Cesare and Co., Rauscedo, Italy), placed in bins of moistened media composed of equal parts perlite and vermiculite, and set in a callus room kept at 27 °C and approximately 80% relative humidity. After two weeks, the grafted vines were transplanted to sleeves containing a mix of equal parts perlite, vermiculite and peat and returned to the callus room until the initiation of shoot growth. The vines were transferred between a fog house, in a greenhouse, and under shade cloth outside for hardening off before being transplanted to the field 23 June 2014. The process was repeated the following year with vines field-transplanted on 18 June 2015. Grafted vines were planted at 0.6 m within row and 3.0 m between rows in a randomized complete block design with one replicate per block. Vines were irrigated using surface drip irrigation. No fertilizer was supplied pre- or post-planting during the first year of vine growth. Second-year vines were supplemented with a surface application of pelleted fertilizer. On 10 February 2016, the shoots were removed from all vines, and intact root systems were excavated using a tractor-drawn U-bar set at 75 cm depth. Roots were immediately washed, drained of excess water, and stored in plastic bags at ~2 °C until analysis.

### 4.3. Analysis of Root Morphology

Root systems were scanned with an Epson Perfection V700 Photo scanner set at 600 dpi, and scanned images were measured using the root image analysis software WinRhizo (Regent Instruments, Inc., Québec, QC, Canada). The software separates roots into user-specified diameter classes and generates root length, surface area, volume, and other values for each diameter class. Based on results from previous experiments, we used different diameter class distributions when analyzing field and greenhouse root systems. For greenhouse-grown root systems, our analysis included roots between 0.0–2.0 mm diameter at 0.1 mm increments to allow adequate resolution in distinguishing among the fine roots of young plants. For field-grown roots in which many of the finest roots are not retained through the winter and during excavation, we measured roots at 1 mm diameter intervals up to 10 mm. Additionally, we measured roots of 0.0–0.5 mm and 0.5–1.0 mm diameters to account for differences in diameter of the finest roots previously observed in field-grown grapevines. Roots were dried at 65 °C and weighed to determine dry biomass.

High plasticity and the heterogeneous nature of soils and growing media result in considerable variation in root systems, even among genetically identical plants. For this reason, absolute values derived from image analysis were used to calculate the proportion of total root length represented by diameter classes, mean root diameter and specific root length (SRL; root length ⋅ dry biomass^−1^), three parameters that are independent of the absolute size of root systems.

### 4.4. Analysis of Root Morphology in Rhizotron Experiment

Images of the root system of each plant were taken every 2–3 days while plants were intact. The last root images of the intact plant were taken on the day of harvest. While examining individual roots, each root was dissected and immediately photographed on a white background before any measurements took place. After dissection, fine roots were placed in containers with water to prevent dehydration. Root length, root diameter, root color, total root length, root angle, cumulative root fraction, numbers of laterals and laterals length were all calculated with Fiji imaging-processing software.

### 4.5. Measurements of Plant Water Status

A Scholander pressure chamber (Soil Moisture Equipment Corp 3005, Goleta, CA, USA) was used to measure stem water potential (Ψ_stem_) of plants in the rhizotron experiment. Mature leaves were placed into aluminum bags for at least 15 min, such that they were hydraulically equilibrated with Ψ_stem_. Subsequently, leaves were excised at the base of the petiole and placed into the pressure chamber, while still bagged. The chamber was pressurized, and the pressure required to force water out of the petiole base was recorded and defined as Ψ_stem_.

### 4.6. Measurements of Soil Water Content (SWC)

Before plants were harvested from rhizotrons, soil samples were taken using a brush (Royal and Langnickel^®^ Essentials™ number 8; Royal and Langnickel, Munster, IN, USA) from different positions along the length of fine roots, then placed in small vials and immediately sealed with a cap to prevent dehydration. The mass of moist soil was measured with a scale a few hours later. Moist samples were placed inside an oven heated to 45 °C temperature for at least two weeks to allow evaporation of all water. Then, when the soil samples were completely dry, the mass of oven-dried soil was measured again. SWC was calculated as:

soil water content (%) = [(mass of moist soil (g) − mass of oven-dried soil (g))/mass of oven-dried soil (g)] × 100,

### 4.7. X-ray MicroCT

X-ray imaging of plant tissue was performed at the Advanced Light Source (ALS) Lawrence Berkeley National Laboratory, beamline 8.3.2. Plants were transported by car from the UC Davis campus to ALS. Upon arriving, Ψ_stem_ was measured immediately. Fine roots were carefully extracted and prepared for imaging following the same protocol of our previous work [11,28,29]. The different sections of roots were mounted into a drill chuck, fixed on an air-bearing stage [28,56,57,58] and imaged using an 18 keV synchrotron X-ray beam. Roots were imaged at the same position were soil samples were taken, indicating the position with a copper wire. During the scanning, 1024 longitudinal images were taken in 180° rotation and 200 s exposure time. Images were collected using a 4008 × 2672-pixel CCD camera (#PCO 4000, Cooke Corporation, Eliot ME, USA). The resolution of the images was 1.8 μm/pixel. The acquired images were reconstructed into a stack of transverse images using Octopus 8.3 software (Institute for Nuclear Sciences), a custom plug-in for Fiji imaging-processing software.

### 4.8. Statistical Analysis

Analysis of variance (ANOVA) and *t*-tests were performed using R v4.0.0 statistical computing environment with aid from the car software package [59]. Where appropriate, the Shapiro-Wilk test and Levene’s test were used to test the assumptions of normality of residuals and homogeneity of variances, respectively. Data were transformed as necessary when assumptions were not met. For the greenhouse, differences between rootstocks were determined using Welch’s *t*-test. Differences in field grown vines were determined with *t*-test. In the analysis of root morphology in the rhizotron experiment, differences in rootstock and drought stress treatment were analyzed using two-way ANOVA and a post-hoc test (honestly significant difference (HSD)-Tukey α = 0.05). Because of the binary nature of the lacunae presence response variable, the predictor variables rootstocks (i.e., 101-14Mgt and 110R), and position (i.e., base, mid-section, a root rip) were modeled with a generalized linear model (glm) using a log link function (‘lme4’ package; Bates et al., 2015). Post-hoc estimates of mean separation were performed using the emmeans package with critical *p* value of 0.05 [60].

## Figures and Tables

**Table 1:** Mean root diameter and specific root length (SRL) in 101-14Mgt and 110R root systems grown from herbaceous cuttings in the greenhouse (*n* = 15) and from dormant woody cuttings grafted to a common scion after one year (*n* = 8) or two years’ (*n* = 5) growth in the field. Values are means ± standard error (SE).

Cultivar	Greenhouse		Field, 1st-Year		Field, 2nd-Year	
	Diameter (mm)	SRL (cm mg^−1^)	Diameter (mm)	SRL (cm g^−1^)	Diameter (mm)	SRL (cm g^−1^)
101-14Mgt	0.63 ± 0.02	3.03 ± 0.16	1.79 ± 0.08	57.8 ± 3.7	1.25 ± 0.06	129.1 ± 17.2
110R	1.06 ± 0.05	1.49 ± 0.09	2.48 ± 0.06	25.4 ± 1.8	2.29 ± 0.08	29.4 ± 2.8
*p*-value	***	***	***	***	***	**

For the greenhouse, differences between rootstocks were determined using Welch’s *t*-test. Differences in field grown vines were determined with *t*-test. *p* < 0.01 = **; *p* < 0.001 = ***.

**Table 2:** Morphological root traits of 110R and 101-14Mgt rootstocks under well-watered (i.e., control) and drought conditions observed in the rhizotron experiment.

Cultivar	Treatment	Angle (°)	Laterals (n)	Root Diameter (mm)	Mean Length of Laterals (cm)	TRL (cm)	Distance from Tip to Nearest Lateral (cm)	White Root Length (cm)	Proportion of Root Length That Is White
101-14Mgt	Control	32.22 ^a^ ± 4.28	81.90 ^ab^ ± 8.79	1.21 ^ab^ ± 0.14	1.75 ^b^ ± 0.15	157.11 ^ab^ ± 20.86	6 ^a^ ± 1.38	2.18 ^ab^ ± 1.26	0.06 ^ab^ ± 0.03
110R	Control	50.01 ^b^ ± 5.07	90.28 ^ab^ ± 7.79	1.91 ^c^ ± 0.14	1.11 ^a^ ± 0.15	95.85 ^ab^ ± 20.86	7.6 ^ab^ ± 1.22	5.71 ^b^ ± 1.12	0.13 ^b^ ± 0.03
101-14Mgt	Drought	41.56 ^ab^ ± 2.62	92.55 ^b^ ± 5.00	1.22 ^a^ ± 0.08	1.51 ^ab^ ± 0.08	144.63 ^b^ ± 11.62	5.5 ^a^ ± 0.78	1.04 ^a^ ± 0.73	0.03 ^a^ ± 0.02
110R	Drought	44.09 ^ab^ ± 2.44	72.18 ^a^ ± 4.44	1.55 ^b^ ± 0.07	1.21 ^a^ ± 0.08	97.65 ^a^ ± 10.89	10.67 ^b^ ± 0.69	5.42 ^b^ ± 0.64	0.13 ^b^ ± 0.02
*p*-value	Rootstock	0.059	0.024	0.001	0.001	0.001	0.001	0.001	0.001
*p*-value	Treatment	0.49	0.419	0.1102	0.568	0.755	0.15847	0.4981	0.7541

Values are mean ± SE. Letters indicate significant differences between groups means within each column as determined by post-hoc test (honestly significant difference (HSD)-Tukey α = 0.05).

**Table 3:** Comparison between the predicted classification performed by the logistic regression model and the observed classification in the experiment using a cut point of 0.5.

	Predicted by the Model	
	Negative (0 = No Lacunae)	Positive (1 = Lacunae)
Observed in the experiment		
Negative (0 = no lacunae)	15.9	15.3
Positive (1 = lacunae)	6.3	62.4

## Data Availability

The data presented in this study are available on request from the corresponding author.

## Supplementary Materials

The following are available online at https://www.mdpi.com/article/10.3390/plants10061121/s1, Figure S1: Root distributions by diameter class in 101-14Mgt and 110R root systems grown from dormant woody cuttings grafted to a common scion after one-year (top; *n* = 8) and two years’ (bottom; *n* = 5) growth in the field. Boxes represent first and third quartiles; bars represent samples within 1.5 times the interquartile range. Although included in statistical analyses, outliers are not displayed. Asterisks indicate significant differences between rootstocks at each diameter class as determined by Welch’s *t*-test. *p* < 0.05 = *; *p* < 0.01 = **; *p* < 0.001 = ***.
