# Supplementary material for: Inherent and Stress-Induced Responses of Fine Root Morphology and Anatomy in Commercial Grapevine Rootstocks with Contrasting Drought Resistance
Source: Plants (Basel). 2021 Jun 1;10(6):1121. doi: 10.3390/plants10061121 (PMC8227383; doi:10.3390/plants10061121)

Supporting Information

Article title: **Inherent and stress-induced responses of fine root morphology and anatomy in commercial grapevine rootstocks with contrasting drought resistance**

Authors: **Idan Reingwirtz[1+], Jake Uretsky[1+], Italo F. Cuneo[2], Thorsten Knipfer[3], Clarissa Reyes[1], M. Andrew Walker[1] and Andrew J. McElrone[1,4*]**

The following Supporting Information is available for this article:

Figure S1. Root distributions by diameter class in 101-14Mgt and 110R root systems grown from dormant woody cuttings grafted to a common scion after one-year (top; n = 8) and two years' (bottom; n = 5) growth in the field. Boxes represent first and third quartiles; bars represent samples within 1.5 times the interquartile range. Although included in statistical analyses, outliers are not displayed. Asterisks indicate significant differences between rootstocks at each diameter class as determined by Welch's t-test. $p < 0.05$ = *; $p < 0.01$ = **; $p < 0.001$ = ***.

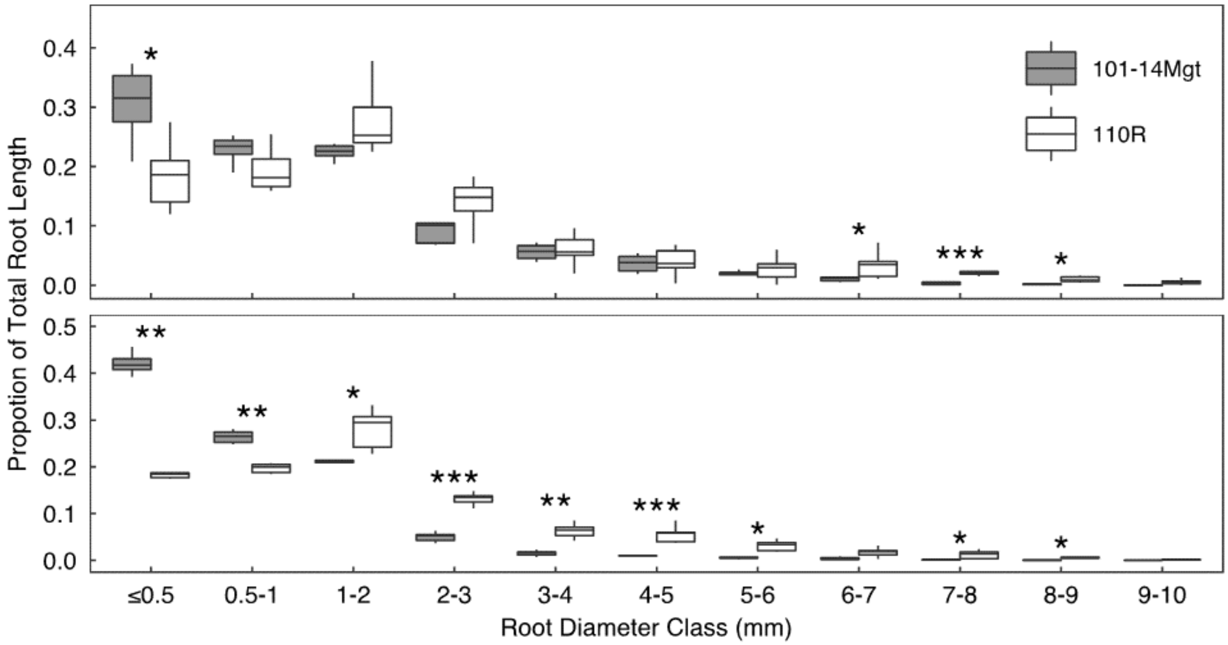

Supplement: Supplementary file 1 [file plants-10-01121-s001.zip › plants-1219806-supplementary.pdf]
